# Fluorescence chromosome banding and FISH mapping in perennial ryegrass, *Lolium perenne* L.

**DOI:** 10.1186/s12864-016-3231-z

**Published:** 2016-11-25

**Authors:** Helal A. Ansari, Nicholas W. Ellison, Shalome A. Bassett, Syed W. Hussain, Gregory T. Bryan, Warren M. Williams

**Affiliations:** 1 AgResearch Ltd, Grasslands Research Centre, Tennent Drive, Private Bag 11008, Palmerston North, 4442 New Zealand; 2 Present address: 16 Moerangi St., Palmerston North, 4410 New Zealand

**Keywords:** *Lolium perenne*, Perennial ryegrass, Chromosome Q-banding, FISH mapping, Chromosome identification, Standardized banded karyotype, Banded ideogram

## Abstract

**Background:**

The unambiguous identification of individual chromosomes is a key part of the genomic characterization of any species. In this respect, the development and application of chromosome banding techniques has revolutionised mammalian and especially, human genomics. However, partly because of the traditional use of chromosome squash preparations, consistent fluorescence banding has rarely been achieved in plants. Here, successful fluorescence chromosome banding has been achieved for the first time in perennial ryegrass (*Lolium perenne*), a forage and turf grass with a large genome and a symmetrical karyotype with chromosomes that are difficult to distinguish.

**Results:**

Based on flame-dried chromosome preparations instead of squashes, a simple fluorescence Q-banding technique using quinacrine mustard, unambiguously identified each chromosome and enabled the development of a banded karyotype and ideogram of the species. This Q-banding technique was also shown to be compatible with sequential FISH mapping enabling labelled genes and molecular markers to be precisely assigned to specific cytogenetic bands. A technique for DAPI-banding, which gave a similar pattern to Q-banding, was also introduced. This was compatible with FISH mapping and was used to anchor a single copy gene from an earlier mapped linkage group of *L. perenne*, thus providing a step towards integration of the genetic and cytogenetic maps.

**Conclusions:**

By enabling the allocation of genes mapped by other methods to physically identified chromosome positions, this work will contribute to a better understanding of genomic structures and functions in grasses.

## Background

The development and application of genomic methods to plant genetics and breeding involves the integration of information across the spectrum from DNA sequences to chromosomes, the units of transmission of genetic information [1]. A complete genomic analysis thus requires an ability to unambiguously identify the individual chromosomes of a species. Before 1970, the only available parameters for chromosome identification were the relative lengths and arm ratios [2]. Such parameters were not sufficient to identify chromosomes in species where chromosomes exhibited gross morphological similarities. The development of chromosome banding techniques in the 1970’s resolved this limitation to a large extent and promoted rapid advances in mammalian and human cytogenetics [3]. The patterns of longitudinal differentiation along chromosomes, produced through G-, Q- or R-banding protocols, are specific for each pair of chromosomes in a given species. This unambiguous chromosome identification has led to the development of standardized banded karyotypes incorporating a chromosome numbering system and banding nomenclature for several mammalian species including human [4], cattle [5], sheep [6] and dog [7]. As modern cytogenetics now incorporates several molecular-based technologies, fluorescence *in situ* hybridization (FISH), which allows direct visualisation of specific DNA sequences on chromosomes, plays an important role in bridging the gap between classical cytogenetics and molecular genetics. The application of a ‘toolbox’ of standardized banded karyotypes in combination with FISH has revolutionised structural, functional and comparative genomics of several mammalian species. On the rare occasions when the chromosome banding pattern cannot resolve chromosome pairs with similar morphologies, FISH mapping of molecular marker sequences can be applied [8].

Similar chromosome banding protocols have not yet been developed for plant species. Several variations of chromosome banding techniques have been tried with plant chromosomes [9–12] but consistent patterns necessary for sequential mapping have not been achieved [13]. It has been argued that the relatively enhanced compactness of the chromatin of plant metaphase chromosomes and/or the adherence of cytoplasmic debris over chromosome preparations obtained from traditional squash preparations obliterates the resolution of G-bands [9, 14]. A fluorescence DAPI (4’, 6-diamidino-2-phenylindole)-banding technique has recently been reported for plant chromosomes which is compatible with FISH mapping experiments using repetitive DNA sequences [15]. The banding patterns were resolved by digital deconvolution and reconstruction of 3D images of chromosomes.

Perennial ryegrass, *Lolium perenne* (2n = 2x = 14) (subfamily Pooideae; tribe Poeae) is native to the temperate parts of Asia, Europe and North Africa. It belongs to the same grass family as several important food crop plants including wheat (*Triticum* spp.), barley (*Hordeum vulgare*), rice (*Oryza sativa*), maize (*Zea mays*), sorghum (*Sorghum bicolor*), as well as the model grass, *Brachypodium distachyon*. It is widely cultivated for forage and turf because of its rapid establishment from seed, high yield, and good digestibility. However, perennial ryegrass has only limited variation for tolerance to biotic and abiotic stresses [16, 17]. Genomics-supported breeding strategies provide a promising approach to the improvement of the agronomic performances and stress tolerances of many crops, including *L. perenne*. The complexity of the perennial ryegrass genome, posed by its large size (2.6 Gbp/1Cx; [18]) and consisting of a significant proportion of repetitive DNA sequences, has hampered progress in sequencing of this genome. Nevertheless, ryegrass genomics is advancing rapidly, with the availability of a draft genome sequence [19] and several linkage maps [20–23]. Despite these advances, it has not so far been possible to unambiguously relate LGs with individual *L. perenne* chromosomes. A key reason is that all seven pairs of conventionally stained chromosomes of ryegrass, biarmed in morphology, display a symmetrical karyotype [24, 25] which makes their individual identification extremely difficult [1].

In this work, we have successfully obtained for the first time fluorescence Q-banding patterns using quinacrine mustard (QM) on ryegrass chromosomes prepared by an improved flame drying technique previously developed in our laboratory [26]. Importantly, the banding method is compatible with the FISH technique for regional chromosomal mapping of DNA sequences so that the banding and the FISH signals can be visualised in the same cell. These results have subsequently led us to produce a banded karyotype for the first time and develop a corresponding banded ideogram for perennial ryegrass. We have used this resource as a tool to map 18S rDNA and 5S rDNA sequences with FISH to precise chromosome bands. In addition, we have mapped a single copy gene to a *L. perenne* cytogenetic band, and so have demonstrated the anchorage of a linkage map marker to a chromosome region. Potential applications are discussed, including the combining of genetic and cytological information into an integrated cytogenetic map.

## Results

**Fig. 1:**
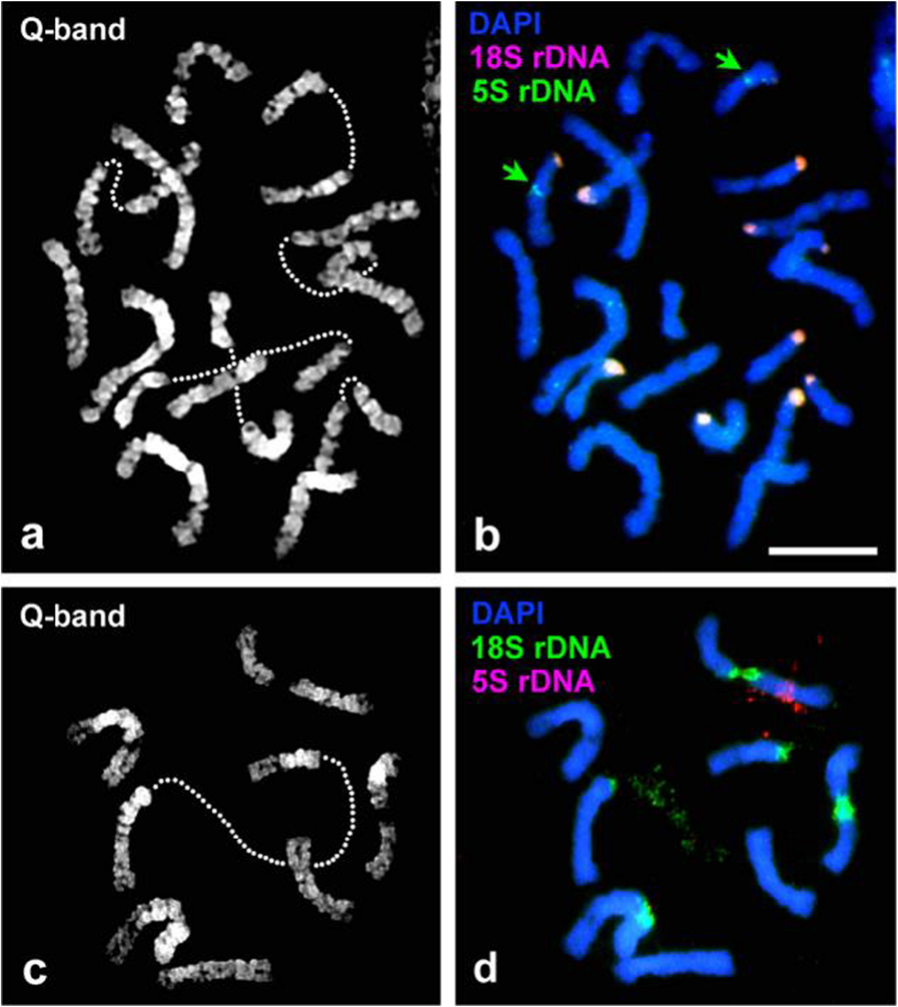
Q-banding and sequential FISH mapping in *L. perenne*. Early metaphase cells of diploid (**a**, **b**) and haploid (**c**, **d**) *L. perenne* after Q-banding (**a**, **c**) and sequential FISH mapping (**b**, **d**) with 5S and 18S rDNA sequences. *Dotted lines* in (**a**) and (**c**) denote decondensed NORs. *Bar* represents 5 μm

**Fig. 2:**
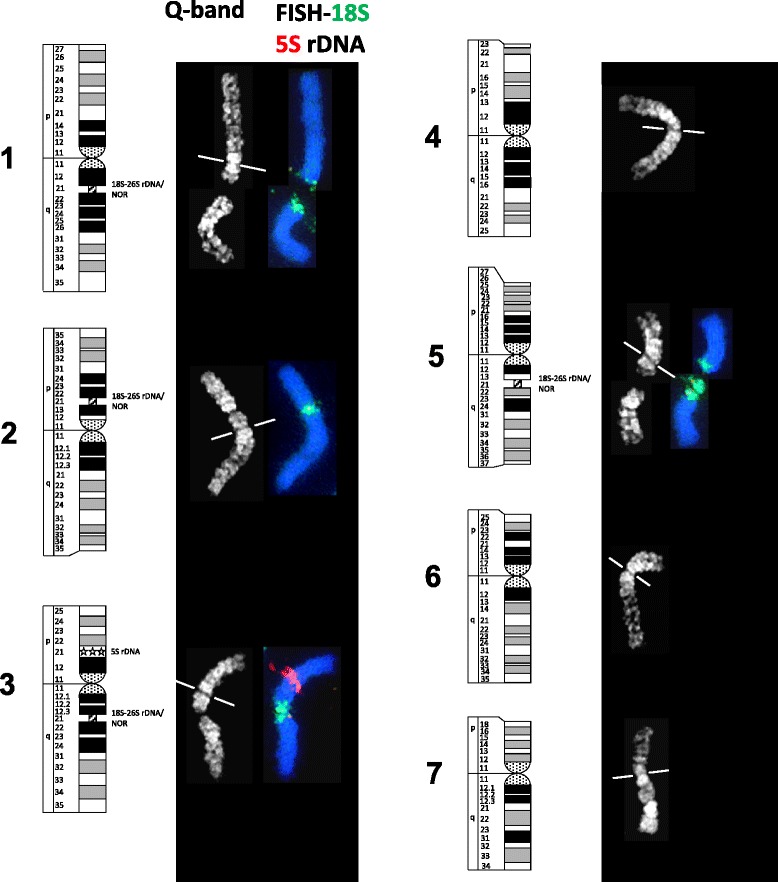
Q-band karyotype standardization of *L. perenne* with sequential FISH mapping of cytogenetic markers, Representative Q-banded karyotype (grayscale) and sequential FISH mapping of 5S (*red*) and 18S (*green*) rDNA sequences. Diagrammatic representation of Q-banding on left of each chromosome. Lines across chromosomes represent centromeric positions

**Fig. 3:**
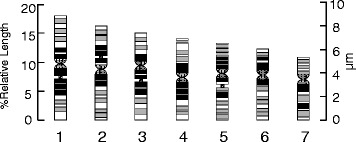
Q-banded ideogram of *L. perenne*

**Fig. 4:**
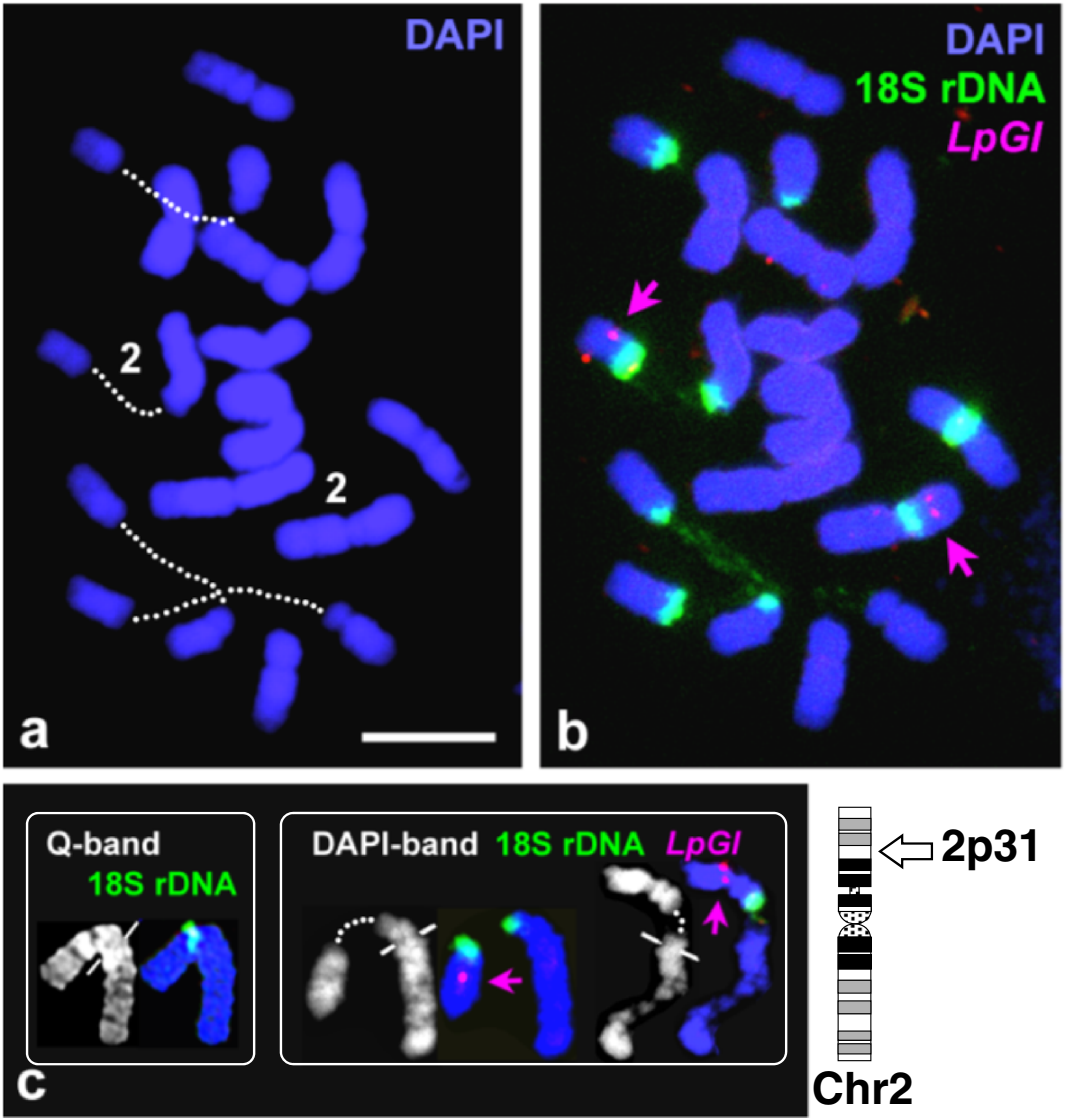
DAPI-banding and FISH mapping of *LpGI* gene in *L. perenne*. Metaphase cell after (**a**) DAPI staining and (**b**) FISH mapping with *LpGI* (*red*) and 18S rDNA (*green*) sequences. In (**c**), the left inset shows an example of Q-banded chromosome 2 (*grayscale*) with sequential hybridization of 18S rDNA. The right inset shows DAPI-banded chromosome 2 in grayscale along with *LpGI* (*red*) and 18S rDNA (*green*) hybridizations from two cells at different condensation levels. At the bottom right, *LpGI* is positioned on the banded ideogram of chromosome 2. *Red arrows* in (**b**) and (**c**) indicate *LpGI* FISH signals. *Lines* across chromosomes in (**c**) represent centromeric positions. *Bar* represents 5 μm

Quinacrine mustard staining displayed distinct and reproducible Q-banding patterns on flame-dried preparations of the somatic chromosomes of both diploid and haploid *L. perenne*. The same preparations were then subjected to FISH procedures to achieve successful regional mapping of 5S and 18S rDNA sequences (Fig. 1). The availability of large numbers of suitable cells allowed us to produce a Q-banded karyotype for *L. perenne* (Fig. 2). Individual chromosome pairs in each cell were identified based on morphology, FISH mapping of rDNA markers and banding patterns. Thereafter, morphometric analysis was carried out based on the 10 selected cells (Table 1). The seven pairs of diploid *L. perenne* chromosomes displayed a gradation in size and have been numbered according to their decreasing size order (Fig. 3). The chromosome sizes ranged from chromosome 1, with 17.93% of the total haploid set to chromosome 7, with 10.83% of the total haploid set. Based on the estimated nuclear genome size of *L. perenne* (2.623 Gbp/1Cx; [18]), the molecular sizes of the chromosomes ranged from 470 Mbp for chromosome 1 to 284 Mbp for chromosome 7. FISH mapping provided marker status to three chromosome pairs in the diploid *L. perenne*. Chromosomes 2, 3 and 5 displayed proximal hybridization of 18S rDNA representing nucleolus organizer regions (NORs) or secondary constrictions. While 18S rDNA hybridized on the long arm in chromosomes 3 and 5, it was localised on the short arm in chromosome 2 (Fig. 2). Among these three NOR carrying chromosomes, chromosome 3, with a secondary constriction on the long arm, co-localized with 5S rDNA on the short arm. In our chromosome preparations, the GC-rich NOR chromatin was often decondensed and occasionally stretched, generally with condensed flanking ends (Fig. 1). Consequently, in DAPI staining the two condensed components of the same chromosome often appeared to be separated, without any visible connection. FISH showed that these parts were joined by cloudy decondensed 18S rDNA (Figs. 1d and 4b) and that there was no chromosome breakage. In some cells the FISH signals of the highly decondensed 18S rDNA were not picked up by the camera, but they were always visible under the microscope.

**Table 1:** Morphometric analysis of *L. perenne* karyotype

Chromosome No.	%L^R^ ± SE^a^	Molecular size of chromosome (Mbp)^b^	I^C^ ± SE^c^	Classification of chromosome^d^
1	17.93 ± 0.30	470.30	46.92 ± 0.45	m
2	16.39 ± 0.28	429.90	46.60 ± 0.53	m
3	15.50 ± 0.24	406.56	36.44 ± 0.96	sm
4	14.07 ± 0.20	369.03	47.68 ± 0.28	m
5	12.94 ± 0.26	339.41	38.21 ± 0.56	m
6	12.30 ± 0.14	322.62	36.60 ± 0.78	sm
7	10.83 ± 0.16	284.07	34.93 ± 0.81	sm

^a^%L^R^ (%relative length) = Length of a chromosome/Total haploid chromosome length x 100

^b^Based on reported genome size estimation [18]

^c^I^C^(centromeric index) = Length of short arm/Chromosome length x 100

^d^As previously reported [2]

The seven chromosomes of the haploid *L. perenne* plant displayed identical Q-banding patterns to those of the seven pairs of the diploid plant. However, instead of three marker chromosomes, four chromosomes displayed 18S rDNA hybridization (Fig. 1) with an additional NOR being detected proximally on the long arm of chromosome 1 (Fig. 2). Regional hybridization of the 18S and/or 5S rDNA sequences on chromosomes 2, 3 and 5 was identical to that observed in the diploid plant.

All seven chromosomes from each cell of the haploid plant were identified and karyotyped as for the diploid cells. An ideogram was drawn based on the data provided in Table 1. The Q-banding patterns for all seven chromosomes were analysed from cells selected from both the diploid and haploid plants. Landmark bands were identified, and the banding patterns and their nomenclature were diagrammatically represented in the banded ideogram with 156 bands (Fig. 3). This enabled us to assign the 5S and 18S rDNA loci in *L. perenne* to a specific chromosome, chromosome arm and band position. The 18S rDNA sequences have been assigned to chromosome positions 1q21, 2p21, 3q21 and 5q21 while 5S rDNA was on 3p21 (Fig. 2).

To test if DAPI-banding on the flame-dried chromosome preparations could also be produced while maintaining compatibility with the FISH procedure, a modified protocol was developed to map a circadian clock-associated gene of *L. perenne*, GIGANTEA (*LpGI*), using genomic as well as cDNA sequences as probes. Although a defined DAPI differential staining was not observed on mid-metaphase chromosomes, low levels of Q-like DAPI-banding were generated in pro-metaphase and early metaphase chromosomes. Specific and identical hybridization of both the probes was observed on a chromosome arm with 18S rDNA. Using the combined banding and marker patterns, *LpGI* was unambiguously assigned to 2p31 (Fig. 4).

## Discussion

A simple fluorescence based Q-banding technique using quinacrine mustard for plant chromosomes, which is compatible with sequential FISH mapping of cloned DNA sequences, is reported here for the first time. This technique enabled the visualization of chromosome banding patterns and FISH signals in the same cell. The longitudinal differentiation of Q-bands produced along the length of each chromosome facilitated unambiguous identification of perennial ryegrass chromosomes. Based on these results, a banded karyotype and ideogram of perennial ryegrass was developed and used as a cytogenomic tool. Based on only two individuals from different cultivars, this represents a first step, and analyses of more populations is needed before a standardized karyotype can be established.

Success was also achieved in producing DAPI-banding patterns while maintaining compatibility with FISH mapping of a single copy sequence on the same cytological preparation. While the DAPI-bands could be visualised only in pro-metaphase and early metaphase cells (Fig. 4), the banding patterns were similar to Q-banding as would be expected because both QM and DAPI are AT-specific fluorochromes [27]. Although less defined than Q-bands, the DAPI bands were sufficiently clear to identify commonality. DAPI-bands were not observed by Rocha et al. [25] who restricted their observations to metaphase cells of *L. perenne*. Cytochemical pre-treatments for the production of bands on fixed chromosomes may cause minor destruction or loss of chromosomal DNA [15, 28]. Such preparations may then be incompatible with FISH mapping, especially with low-copy targets. Because the Q- or DAPI-banding techniques presented here did not involve any specific pre-treatment before staining with QM or DAPI, they were compatible with FISH mapping.

Technical limitations in chromosome preparation have previously hampered progress in developing plant chromosome banding techniques. The traditional squash method causes adherence of cytoplasmic debris from clumped meristematic tissue and may have been the main barrier to generating resolvable bands on plant chromosomes [9, 29]. The flame-drying method reported previously [26], and adopted in this work, did not involve the traditional squashing of tissue. Instead, the enzymatically macerated meristematic tissue was teased into a droplet of cell suspension, given a temperature shock with chilled fixative and then flame-dried. This left the chromosomes well-spread and completely clean on the glass slide without any cytoplasmic debris. An additional advantage of the well-spread and debris-free chromosome preparation has been the enhanced sensitivity of FISH signals [30, 31]. Here, we have successfully mapped a small target sequence, *LpGI*, onto a specific chromosome band of *L. perenne* using both genomic DNA and cDNA probes.

Variability in the number of NOR loci in *L. perenne* has been described in earlier reports [25, 32–34]. Six NOR loci have been common and an additional NOR FISH signal has been recorded in a few reports [25, 33, 34], but eight NOR loci have not been encountered. The chromosomes carrying these sequences were either not numbered or were inconsistently numbered. In the present work, the three pairs of chromosomes in the diploid *L. perenne* plant consistently carrying NOR loci were designated according to the proposed karyotype. The NOR (18S rDNA) loci and the single 5S rDNA locus were then regionally mapped and assigned to cytogenetic bands. Identical assignments were made in the haploid *L. perenne* but with the addition of a fourth 18S rDNA signal on the largest chromosome. This was consistent with the previous observation [25] that a hemizygous 18S rDNA locus occurred on the largest chromosome.

Seven pairs of biarmed chromosomes of *L. perenne*, were numbered according to decreasing size order in the karyotype presented here. A combination of gross chromosome morphology, Q-banding patterns and molecular cytogenetic markers (two rDNA sequences) has helped in individualizing all seven pairs for the first time (Fig. 2). An estimation of the molecular size of each chromosome has also been presented, based on the nuclear genome size determined earlier by flow cytometry [18]. The chromosome designations given here (Table 1) are similar, but not identical, to those assigned by Rocha et al. [25]. Chromosomes 1–5 are probably the same in both classifications, but the potential confusion of chromosomes 6 and 7, which are of similar morphology and size, can now be resolved by Q-bands.

*L. perenne* exhibits extensive conserved synteny with the *Triticeae* and other members of subfamily Pooideae [21, 35]. Various gene maps of *L. perenne* have been developed through comparative mapping, mainly with *Triticeae* [20–22]. The numbering system of linkage groups (LGs) in these maps have directly been adapted from *Triticeae* [21, 22, 36, 37]. Due to the lack of a standardized karyotype of *L. perenne*, no correlation between LGs and individual chromosomes has been established so far through FISH mapping. Neither the analysis of alien substitution lines [38] nor alien introgression lines [39] has provided unambiguous identification of all *L. perenne* chromosomes. Indeed, as noted by Kopecky and Studer [1], only one of the seven chromosomes had so far been discriminated. GIGANTEA (*LpGI*) has been mapped to LG3 of *L. perenne* [40]. Here, this single copy gene has been precisely assigned through FISH mapping to a cytogenetic band on the short arm of chromosome 2 (Fig. 4). LG3 has been intensively analyzed by introgression mapping and the presence of an NOR as noted previously [41], was also found in the present work. Thus Q-banding combined with FISH mapping has enabled LG3 to be identified as chromosome 2 and described by its size, banding pattern and morphology. The accumulation of many such regional assignments should lead to the development of an integrative cytogenetic map. Such an integrative cytogenetic map will provide an alternative approach for analyses of recombination (cM) along the chromosome [39] and reveal the extent of unmapped genomic regions. It could also help to resolve contradictory chromosome assignments of markers [21] and LG map discrepancies [22]. Chromosomal mapping will also allow the placement of markers which cannot be genetically mapped [42].

To facilitate DNA sequencing of large grass genomes, individual chromosomes can be isolated by flow cytometry [31, 43, 44]. DNA preparations from flow-sorted chromosomes can then be used for the generation of chromosome-specific sequences and assemblies [45, 46]. Application of the chromosome banding technique presented here should provide unambiguous identification of flow sorted chromosomes and help to reveal contamination. Flow-sorted chromosomes can also be identified by FISH if appropriate markers are available [18]. However, the chromosome banding method is faster, cost effective and will also reveal the intact status of the sorted chromosomes.

Introgression of alien chromosome or chromosome segments have produced cytogenetic stocks of addition or substitution lines in a number of Pooideae, including *Festulolium* hybrids [38, 47, 48]. Application of the Q-banding technique in combination with sequential FISH and/or genomic *in situ* hybridization (GISH) should provide identification of specific chromosomes and, by extension, the nature and extent of introgression.

Transgenesis is often used by agricultural biotechnologists to introduce traits of agronomic interest into crops [49–54] but instability in the expression of the introduced transgene is common [55–57]. The transgenes may unpredictably integrate at different locations in the host genome and be subject to “position effect” variation in expression patterns [51, 58]. The chromosome banding technique developed here with the concomitant visualization of the integrated transgene through FISH should reveal the number of insertion events and the hemi- or homozygosity of each insertion. Further, the precise cytogenetic determination of transgene integration site(s) may throw light on the relationship between transgene expression pattern and the location of integration. Additionally, it should detect tissue culture-mediated chromosomal abnormalities which frequently occur in transformed plants [59, 60].

## Conclusions

The Q-banding method described here has enabled the unambiguous identification of all seven chromosomes (and all 14 chromosome arms) of *L. perenne*. Further, the technique has been demonstrated to be compatible with FISH mapping, enabling the anchoring of a locus mapped by linkage analysis to a specific chromosome position on an identifiable chromosome arm. Similar work should quickly enable the anchoring of many more loci currently located by linkage analysis to specific chromosome positions, thus integrating cytogenetics and genetics and enabling LGs to be associated with identifiable chromosomes. Extension of the method to the close relatives of *L. perenne* is likely to greatly facilitate the development of addition/substitution lines and, by extension, the integration of introgression bins with known chromosome positions. The work thus provides an integrative cytogenomic resource that will contribute to a better understanding of genomic structures and functions in grasses.

## Methods

### Plant materials and chromosome preparation

Diploid perennial ryegrass (accession no. A10642, a late-flowering selection of New Zealand x Spanish origin) and haploid perennial ryegrass derived from anther culture [61] of cultivar Option WH-1, were obtained from the Margot Forde Forage Germplasm Centre at AgResearch Grasslands, Palmerston North, New Zealand and grown under glasshouse conditions. Actively growing root tips from single individuals of each were processed for somatic chromosome preparations according to the flame drying technique described previously [26] with minor modifications. Briefly, freshly harvested root tips were incubated with 3 mM 8-hydroxyquinoline for 2 h at 22 °C and then for 6 h at 4 °C. These were fixed in 3:1 methanol-acetic acid solution and stored at 4 °C. Root tips were washed in citrate buffer (4 mM citric acid, 6 mM sodium citrate, pH 4.8) and then macerated in 2% (w/v) cellulase (1.6% cellulase Calbiochem 515883 + 0.4% cellulase Onozuka R-10) in citrate buffer at pH 4.8 and 20% (v/v) pectinase (from *Aspergillus niger* in 40% glycerol, Sigma P-0690) for from 50 min to 1 h at 37 °C. After washing in citrate buffer, root tips were placed on a clean glass slide in a droplet of the buffer and apical meristematic tissue was gently extruded from the surrounding tissues under a stereomicroscope. Excess buffer was aspirated out, the meristematic tissue was dissociated into a droplet of cell suspension using fine needles and, thereafter, 1 drop of 48% acetic acid was placed on the cell suspension. After 2 min of incubation at room temperature, 2–3 drops of chilled methanol-acetic acid (3:1) fixative (stored at -20 °C) was gently placed on the slide and quickly flame-dried. Slides were screened using phase contrast optics to assess the quality of cytological preparations.

### Chromosome banding, FISH mapping and karyotype analysis

For Q-banding, cytological preparations were stained with 0.002% quinacrine mustard for 30 s, rinsed with distilled water and mounted in M/15 Sorensen phosphate buffer at pH 6.8. Photomicrographs were taken immediately using appropriate Nikon filters. Coverslips were removed and the slides were rinsed with distilled water and incubated in methanol:acetic acid fixative. After air drying, the slides were ready for sequential FISH procedures.

Three DNA sequences were used as probes for FISH mapping. pTr18S (GenBank accession no. AF071069), a 1.8 kb fragment from *Trifolium repens* containing almost the entire 18S rDNA sequence representing the nucleolus organizer region (NOR), and pTr5S (GenBank accession no. AF072692), a 596 bp fragment encoding *T. repens* 5S rDNA, were labelled with either Fluor-X-dCTP (Amersham) or Cy3-dCTP (Amersham), using nick translation according to the manufacturer’s specifications. A 5.5 kb genomic fragment encompassing a circadian clock-associated gene of *L. perenne*, GIGANTEA (*LpGI*) (GenBank accession DQ534010) and its cDNA complement of 3.44 kb were labelled with Cy3-dCTP.

Sequential double target FISH using the 5S and 18S rDNA probes was performed according to a previously reported method [26] on slides previously used for Q-banding. Here, counter staining of chromosomes subjected to sequential FISH was carried out using DAPI (1 μg/ml in McIlvaine’s buffer, pH 7) for 6 min before mounting in Vectashield (Vector Laboratories). The whole process facilitated visualisation of banding as well as probe localisation in the same cell, allowing regional assignments of DNA probes in relation to chromosome bands. FISH mapping of each *LpGI* probe was performed on separate preparations in combination with 18S rDNA according to the earlier protocol except that these preparations were not subjected to QM staining. In this case, after the FISH procedures, DAPI-banding was achieved by staining the chromosomes with DAPI (0.5 μg/ml in McIlvaine’s buffer, pH 7) for 30 s before mounting in Vectashield (Vector Laboratories). Photomicrographs were taken using a Zeiss monochrome CCD camera on a Nikon epifluorescence microscope Microphot-SA and images were processed with ISIS FISH Imaging System (MetaSystems, Germany).

Ten well spread and good quality Q-banded prometaphase to mid-metaphase cells from each of the diploid and haploid plants were selected for analysis. After chromosome identification based on gross morphology, banding patterns and molecular markers, individual karyotypes were prepared. Morphometric analysis (Table 1) based on 10 cells selected from the diploid plant was carried out according to an established method [2] The banded ideogram was drawn based on morphometric data (Table 1). Chromosomes were arranged in decreasing size order. Relative staining intensity of the bands was represented in the ideogram using one of the three shades from white (no fluorescence), light stipple (medium fluorescence) to black (bright fluorescence). The designation of landmarks and nomenclature of chromosomal regions and bands followed the recommendations of ISCN (1985) [4] for human chromosomes. Briefly, short and long chromosome arms were designated p and q, respectively. Chromosome arms were divided into regions and bands which were numbered outward from the centromere. Consistent and conspicuous banding landmarks were used for delineating the regions. A landmark band was designated as the first band of the region next to the landmark. Each region was divided into bands depending on the staining patterns of the region. A specific chromosomal band was designated by the chromosome number from the standardised karyotype, the arm symbol, the region number and the band number within that region written in continuation without spaces. Occasionally, bands were subdivided at prometaphase or late prophase stages. In these cases, decimal points were used to designate the sub-bands.
